# FRAGMATIC: A randomised phase III clinical trial investigating the effect of **fragm**in^® ^**a**dded to standard **t**herapy **i**n patients with lung **c**ancer

**DOI:** 10.1186/1471-2407-9-355

**Published:** 2009-10-06

**Authors:** Gareth O Griffiths, Sarah Burns, Simon I Noble, Fergus R Macbeth, David Cohen, Timothy S Maughan

**Affiliations:** 1 Wales Cancer Trials Unit, School of Medicine, Cardiff University, Cardiff, UK; 2 Wales Cancer Trials Unit, Velindre NHS Trust, Cardiff, UK; 3 Department of Palliative Medicine, School of Medicine, Cardiff University, Cardiff, UK; 4 Centre for Clinical Practice, National Institute for Health and Clinical Excellence, London, UK; 5 Health Economics and Policy Research Unit, University of Glamorgan, Pontypridd, UK

## Abstract

**Background:**

Venous thromboembolism (VTE) occurs when blood clots in the leg, pelvic or other deep vein (deep vein thrombosis) with or without transport of the thrombus into the pulmonary arterial circulation (pulmonary embolus). VTE is common in patients with cancer and is increased by surgery, chemotherapy, radiotherapy and disease progression. Low molecular weight heparin (LMWH) is routinely used to treat VTE and some evidence suggests that LMWH may also have an anticancer effect, by reduction in the incidence of metastases. The FRAGMATIC trial will assess the effect of adding dalteparin (FRAGMIN), a type of LMWH, to standard treatment for patients with lung cancer.

**Methods/Design:**

The study design is a randomised multicentre phase III trial comparing standard treatment and standard treatment plus daily LMWH for 24 weeks in patients with lung cancer. Patients eligible for this study must have histopathological or cytological diagnosis of primary bronchial carcinoma (small cell or non-small cell) within 6 weeks of randomisation, be 18 or older, and must be willing and able to self-administer 5000 IU dalteparin by daily subcutaneous injection or have it administered to themselves or by a carer for 24 weeks. A total of 2200 patients will be recruited from all over the UK over a 3 year period and followed up for a minimum of 1 year after randomisation. Patients will be randomised to one of the two treatment groups in a 1:1 ratio, standard treatment or standard treatment plus dalteparin. The primary outcome measure of the trial is overall survival. The secondary outcome measures include venous thrombotic event (VTE) free survival, serious adverse events (SAEs), metastasis-free survival, toxicity, quality of life (QoL), levels of breathlessness, anxiety and depression, cost effectiveness and cost utility.

**Trial registration:**

Current Controlled Trials ISRCTN80812769

## Background

### Lung cancer and thromboembolism

In the UK about 33,000 patients are diagnosed with lung cancer every year, with a median 1-year survival of 20% [1]. The death rate from lung cancer in the western world ranges from 5 to 27 per 100,000 per year in women and 25 to 77 per 100,000 per year in men [2,3]. Therapeutic advances over the last 30 years have had only a modest impact on overall survival and there is unlikely to be a marked improvement in survival rates in the coming years even in patients with resectable disease. There is therefore a clear need to investigate new approaches [4].

Venous thromboembolism (VTE) is common in patients with any cancer and the incidence is increased by surgery, chemotherapy, radiotherapy and disease progression [5-8]. This results in a prevalence of clinically apparent VTE of up to 15% [9] across all cancer patients and over 50% of palliative care inpatients [10]. Analysis of US Medicare data suggests that the risk varies with tumour type [11]. Comparable figures are not available specifically for lung cancer patients, but because many have advanced disease at presentation, the prevalence of VTE is likely to be high. Many patients with lung cancer also have co-morbidities, such as heart failure and chronic lung disease, which also increase the risk of VTE by 20% [12].

Buccheri et al [13] studied 286 consecutive patients with lung cancer and found that pre-treatment abnormalities of coagulation were significantly correlated with survival. Large retrospective studies have also shown that the probability of death within 6 months in cancer patients with VTE is 0.94, compared to 0.4 in those without VTE (p = 0.001) [6]. Seitz and co-workers found that lung cancer patients with increased levels of thrombin-anti-thrombin complex (TAT complex) in plasma seemed to have a poorer prognosis than patients with normal levels [14]. Registry data also show that 1-year survival in cancer patients with a VTE is 3 times less than in those without VTE [15].

Low molecular weight heparin (LMWH) has been used for over 20 years in the prophylaxis of VTE and has been shown to be the drug of choice in the treatment of VTE in cancer patients [16]. However, there are limited data on the use of LMWH in primary thromboprophylaxis in cancer patients and this is reflected in the difference in practice amongst oncologists [17]. A recent survey showed that more than 25% of British oncologists do not recognise the thrombogenic effects of treatment for cancer and that thromboprophylaxis is rarely used [18].

In view of the known pro-thrombotic state of lung cancer and the potential antitumour effects of LMWH there is a clear need to assess the impact that long term LMWH has on overall survival in lung cancer patients.

### Prothrombotic state in cancer

Virchow's triad states that conditions predisposing to thrombosis include pathological changes in blood flow, the vessel wall, and coagulability of the blood--all three of which exist in patients with cancer. Proposed mechanisms to explain the hypercoagulability of the blood associated with cancer include both non-specific factors related to the host's response to the tumour and tumour cell-specific causes. Non-specific factors include altered coagulation factor levels and activity, new blood vessel formation (angiogenesis and neovascularisation of the growing tumour mass), cell necrosis, and altered haemodynamics related to obstruction of vessels by the tumour itself. Examples of factors related to properties specific to the transformed cell include expression of activators of thrombin-generating and plasminogen activator-initiated enzymatic pathways, and interaction with host cells, including monocytes, platelets, and endothelial cells [19,20].

Most patients with cancer have blood coagulation test abnormalities indicative of up-regulation of the coagulation cascade, increased platelet activation and aggregation, and increased proteolysis. The complex mechanisms responsible for this activation may include release of cancer procoagulants, activation of host cells (such as monocytes or endothelial cells), overexpression of plasminogen activator inhibitor-1 (PAI-1), and altered expression or activity of proteins produced by the liver, including protein C and anti-thrombin (AT) [20,21].

The best characterised tumour cell initiator of the coagulation cascade is tissue factor (TF), a cell membrane receptor for coagulation factor VII that triggers the extrinsic coagulation pathway by formation of the TF/VIIa/Xa complex. A less well characterised tumour cell activator of coagulation is cancer procoagulant, a cysteine protease that activates factor X. Tumours characteristically promote excessive or unregulated angiogenesis by stimulating the activation, adhesion, migration, proliferation, and transmigration of endothelial cells across tissue matrices. The numerous interactions between coagulation activation and angiogenesis indicate that the latter is an integral part of the prothrombotic state in cancer [20,22-24].

Chemotherapeutic agents may also contribute to hypercoagulability by enhancing the release of procoagulants and cytokines from tumour cells, producing toxic substances, such as oxygen free radicals, that can damage the endothelium, and reducing levels of natural anticoagulants, such as proteins C and S, and AT. Surgery, which is first-line therapy for 15-20% of lung cancer patients, is also well known to activate the haemostatic system. In so doing, cancer therapies initiate the coagulation cascade, promoting thrombosis and tumour growth and producing unfavourable outcomes [8,19].

### Effects of heparins on cancer

A growing body of data suggests that adjunctive therapy with heparin may improve prognosis in cancer patients. Important information has been derived from studies on the treatment of VTE in the general population with different types of anticoagulants. The standard treatment of VTE is a course of heparin followed by an oral vitamin K antagonist. Considering the subgroup of cancer patients with VTE, a meta-analysis found an improved survival in cancer patients treated initially with LMWH when compared with those treated with unfractionated heparin (UFH) [25]. In the 629 cancer patients treated (306 LMWH vs 323 UFH), the pooled odds ratio for 3 month mortality was 0.61 (95% confidence interval 0.4-0.93) in favour of LMWH. In these studies a wide variety of cancer subtypes were present. While this trial did not control for important cancer prognostic variables, such as tumour type and standard treatment, it provided an impetus for researchers to continue investigating heparin's potential as an antineoplastic agent.

A randomised clinical trial of heparin as anticancer therapy was conducted with 277 patients with small cell lung cancer (SCLC) by Lebeau et al [26]. The researchers reported that the complete response (CR) rate was significantly greater in the group receiving chemotherapy plus subcutaneous unfractionated heparin (UFH), compared with chemotherapy alone (37% vs. 23%, respectively; P = 0.004). The median survival was also significantly longer in the UFH group (317 vs. 261 days, respectively; P = 0.01).

The effectiveness of LMWHs in SCLC was evaluated more recently by Altinbas et al [27]. In this trial, 84 patients with SCLC were randomised to receive either chemotherapy (cyclophosphamide, epirubicin, and vincristine, (CEV) or CEV plus LMWH (dalteparin)). At 18 weeks, the overall response rate was higher in patients receiving LMWH plus chemotherapy, compared with chemotherapy alone (69.2% vs. 42.5%, respectively; P = 0.007). Moreover, the duration of progression-free survival was significantly longer with the addition of LMWH (10 months vs. 6 months with chemotherapy alone). This was a small study of only 84 patients and the survival rate in the control arm to chemotherapy was very low.

FAMOUS was the first prospective, randomised, placebo-controlled trial to examine possible effects of LMWH therapy (dalteparin) on survival in patients with various cancers without underlying thrombosis [28]. A total of 382 patients were randomised to receive either 5000 anti-Xa units of dalteparin daily (via injection) or placebo (saline) along with standard cancer therapies. Survival estimates for the dalteparin and placebo group patients at 1 year were 46% (95% CI, 39% to 53%) and 41% (95% CI, 34% to 49%), respectively (p = 0.19). The survival rate at 2 years after randomisation was 27% (95% CI, 20% to 34%) for patients receiving dalteparin versus 18% (95% CI, 11% to 25%) for patients receiving placebo. At 3 years the survival rate was 21% (95% CI, 14% to 28%) for patients in the dalteparin group and 12% (95% CI, 5% to 19%) in the placebo group. However, analysis of a group of patients (not defined a priori) with a better prognosis and who survived beyond 17 months found that LMWH treatment was associated with a significant increase in survival at 25 and 36 months. The investigators concluded that while LMWH therapy did not produce an immediate survival benefit, it may offer an advantage in "better prognosis" patients suggesting an effect on disease progression or metastatic spread.

### Antitumour effects of heparins on cancer

A variety of mechanisms have been proposed to explain the effects of heparin on tumours [20,24,29-32]. These mechanisms can be broadly classified as direct antitumour effects, antiangiogenic effects, and immune modulatory effects. Therapeutic heparin may influence tumour cell growth because of its chemical resemblance to cell surface-associated and extracellular matrix (ECM) heparin-like glycoaminoglycans. These molecules regulate the way in which cells perceive their environment by interacting with growth factors, enzymes, chemokines, and matrix proteins at the interface of the cell and extra cellular matrix so as to modulate signal transduction and thereby regulate malignant cell growth. Under certain experimental conditions, heparin may also induce apoptosis and differentiation of neoplastic cells. Work in experimental models has demonstrated that heparin regulates the expression of certain oncogenes, including c-myc and c-fos [33,34].

The antiangiogenic effects of heparin probably play a central role in its potential use as an antineoplastic agent. These effects appear to be unrelated to the anticoagulant actions of the compound. The evidence suggests that heparin acts by stabilising angiogenic growth factors stored in the ECM.

The effects of heparin on the coagulation cascade include the inhibition of factor IIa, inhibition of TF expression, and activation of Tissue Factor Pathway Inhibitor (TFPI), which down-regulates the activity of TF and factors VIIa and Xa (the extrinsic coagulation pathway). In addition, heparin promotes the release of tissue plasminogen activator and PAI-1 from endothelial cells, facilitating fibrinolysis.

There is great interest in exploring the effects of LMWHs on blood vessels and this interest has focused on the role of TFPI in both thrombosis and noncoagulant processes. A number of preclinical investigations have begun to describe the broad range of TFPI actions. Some of those studies are described below.

Vascular endothelial growth factor (VEGF) is a potent proangiogenic molecule produced by a variety of cell types. Tissue factor pathway inhibitor can modulate the metastatic processes induced by pathological angiogenesis, by blocking both the coagulant and noncoagulant activities of the TF/VIIa/Xa complex [20,24,35]. In a study using a chick chorioallantoic membrane (CAM) model of human colon cancer, the administration of tinzaparin 24 hours after stimulation of angiogenesis by VEGF returned the angiogenesis index to levels comparable to untreated controls [20].

Similar effects of tinzaparin were observed when colon carcinoma (HCT-116) was used to stimulate angiogenesis. Additional research showed an approximately 80% reduction in the growth of colon cancer and fibrosarcoma 1 week after giving a single 0.1 mg dose of tinzaparin in the CAM tumour implant model [36]. The investigators concluded that tinzaparin may play an important role in slowing tumour growth and metastasis by stimulating the production of endothelial TFPI.

A third study looked at platelet count, a sensitive marker of intravascular coagulation and useful indicator of tumour-induced clotting activation. Using an animal model of lung metastasis, Mousa et al observed that the number of platelets fell rapidly and significantly (by nearly 50%) after the injection of tumour cells [20]. This effect was almost completely reversed by tinzaparin. With daily administration of tinzaparin over the next 14 days, the number of lung metastases was reduced by 96% relative to controls. No bleeding problems were observed.

The different antitumour effects of LMWH are likely to vary according to the molecular weight of the drug. Endothelial cell proliferation inhibition increases as the size of the molecule decreases, with optimal effects between 4.5 and 6.5 kDa [35], whilst TFPI release increases with an increase in molecule size [37].

### Choice of heparin and dose in the trial

Dalteparin is a low molecular weight heparin with antithrombotic properties demonstrated by enhancing the inhibition of Factor Xa and thrombin, by anti-thrombin. It potentiates preferentially, the inhibition of coagulation Factor Xa, while only slightly affecting activated partial thromboplastin time (APTT). It is commercially available in over 40 countries. As well as its anticoagulant and antithrombotic properties, dalteparin has been shown to inhibit tumour metastases in in vitro and in vivo studies. Dalteparin is known to inhibit heparinase, an enzyme that is secreted by cancer cells and takes part in the degradation of the extracellular matrix. Heparinase activity correlates with the metastatic potential of mammary adenocarcinoma cells and other cell lines [34,35,37-39].

Since there is no clinical information about dalteparin dosing and antitumour effects, the dose of dalteparin in a study evaluating survival and prophylaxis of thromboembolism should be based on the overall safety profile and efficacy in thromboprophylaxis. The incidence of thrombosis in cancer patients is variable and appears to be dependent on a number of factors including diagnosis, stage of disease, and therapy administered. Thromboprophylaxis with dalteparin 5,000 IU once daily is approved in many countries in high-risk patients undergoing surgical procedures and acutely unwell medical patients. In one study of surgical prophylaxis in patients with cancer, a dose of 5,000 IU was superior to 2,500 IU in cancer patients in terms of thromboprophylactic efficacy and was not associated with an increased risk of severe bleeding [40]. A dose of 5000 IU/day, therefore, has demonstrated efficacy with an acceptable safety profile for patients with lung cancer.

### Main research question

The main study aim of the FRAGMATIC trial is to assess the effect on overall survival of adding Low Molecular Weight Heparin, dalteparin (FRAGMIN^®^) for 24 weeks to standard treatment for patients with lung cancer. We will also examine the impact of dalteparin on venous thrombotic event (VTE) free survival, serious adverse events (SAEs), metastasis-free survival, toxicity, quality of life (QoL), levels of breathlessness, anxiety and depression, cost effectiveness and cost utility. In this paper we describe the study protocol.

## Methods/Design

### Study Design

**Figure 1:**
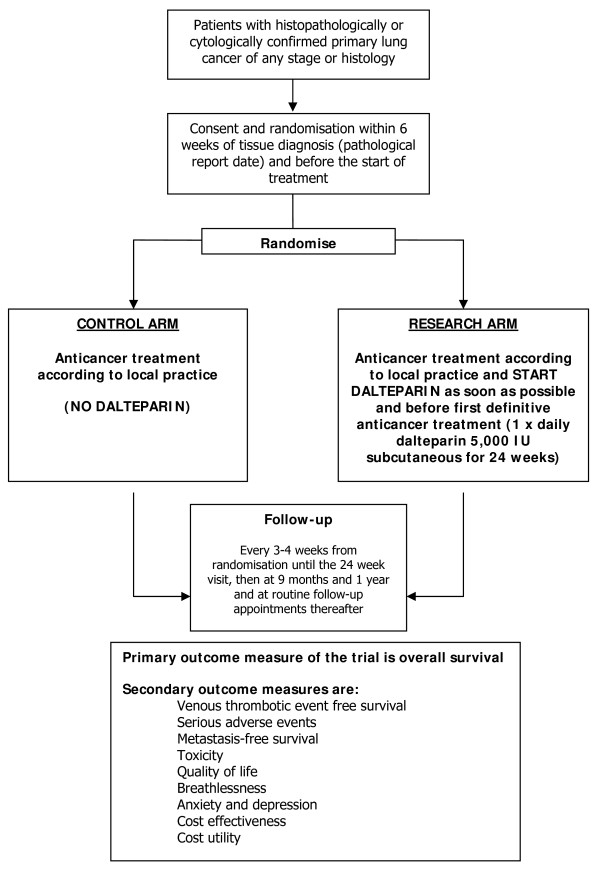
**Trial Schema**.

FRAGMATIC is an open-label two arm randomised controlled trial. It is being run in approximately 120 participating centres throughout the UK with the aim of recruiting 2200 participants, 1100 participants in each arm. At randomisation participants are assigned to either the control arm (no dalteparin) or the research arm (daily dalteparin). See Figure 1. All participants will receive standard anticancer treatment of any type including supportive care. Participants on the research arm will also receive a daily dose of 5000 IU (0.2 ml) of dalteparin for 24 weeks, which will be injected subcutaneously by the participant or a nominated carer.

FRAGMATIC has been ethically approved by the Research Ethics Committee for Wales and has approval from the Medicines and Health Care Product Regulatory Agency to be conducted in the UK. The Wales Cancer Trials Unit, a Cancer Research UK core funded and National Cancer Research Institute accredited Clinical Trials Unit, is coordinating the trial. Velindre NHS Trust is the sponsor for the trial. A Trial Steering Committee and an Independent Data Monitoring Committee has been set up to monitor the progress and safety of the study. The FRAGMATIC Trial Management Group, including clinicians, clinical trial unit staff, patient representatives, nursing and pharmacy representatives, carry out the day-to-day running of the trial.

### Participant Eligibility

Eligible participants are approached within a hospital setting within 6 weeks of their lung cancer diagnosis. Participants are screened for eligibility by research staff to ensure all inclusion and exclusion criteria are met. For inclusion and exclusion criteria see Table 1. Before randomisation participant eligibility is confirmed within 6 weeks of their diagnosis with histology or cytology, and a formal staging assessment. A full blood count, urea, electrolytes, liver function test, corrected calcium, creatinine and clotting profile will be performed 2 weeks before randomisation. After written informed consent has been obtained a baseline assessment of toxicity and dyspnoea score is taken by research staff and participants must complete the baseline Quality of Life and Hospital Anxiety and Depression Scale (HADS) questionnaires. Participants are then randomised to one of the trial arms.

**Table 1:** Inclusion and exclusion criteria for the FRAGMATIC trial

Inclusion Criteria
Patients meeting the following criteria can be included in the trial:
1. Histopathological or cytological diagnosis of primary bronchial carcinoma (small cell or non-small cell) within the last 6 weeks

2. Age 18 or over

3. ECOG Performance status 0, 1, 2 or 3

4. Willing and able to self-administer LMWH by daily subcutaneous injection or have it administered to them by a carer

5. Willing and able to give informed consent.

**Exclusion Criteria**
If any of the following criteria apply, patients cannot be included in the trial:

1. Patients with other intrathoracic tumours (e.g. carcinoid, mesothelioma, lymphoma, lung metastases from another primary site)

2. Any previous illness or treatment likely to interfere with protocol treatment or comparisons

3. Clinically apparent brain metastases

4. Patients who have had a haemorrhagic stroke in the last 3 months

5. Haemoptysis of CTC Grade 2 (symptomatic haemoptysis requiring medical intervention) or above

6. Known bleeding disorder

7. Known pregnancy or lactation. Effective contraception is essential for all female patients (of reproductive potential) if sexually active

8. Known hypersensitivity to dalteparin or other low molecular weight heparins and/or heparins (e.g. history of confirmed or suspected immunologically mediated heparin induced thrombocytopenia; acute gastroduodenal ulcer; subacute endocarditis)

9. Platelet count lower than 100 × 10^9^/L

10. Renal impairment with serum creatinine greater than 150 μmol/L

11. Patients who are currently receiving or have received therapeutic anticoagulation in the last 12 months

12. Patients taking ketorolac (toradol^®^) - this is a non steroidal anti-inflammatory drug (NSAID) with a well documented risk of causing increased bleeding when given with LMWH

13. Patients who at the time of randomisation have a central venous catheter in place and the local practice specifies the use of thromboprophylaxis

14. Any other active malignancy in the last 5 years, except completely treated non-melanoma skin cancer or in-situ carcinoma of cervix. Patients with previous malignancies in remission for at least 5 years can be included, provided that there is a clear MDT decision that this is a new primary.

### Sample Size Considerations

This study aims to demonstrate that dalteparin will improve the 1-year survival of lung cancer patients by 5%. Although the median 1-year survival for all lung cancer patients in the UK has been shown to be 20% [1], it is likely that those with a very short life expectancy will not be entered into this study. For this group of patients it is assumed that the 1-year survival in the control group is 25%. To detect an advantage in 1 year survival of 5% with dalteparin (to 30%) a hazard ratio of approximately 0.87, using a 2-sided log rank test with 89% power at a 5% significance level, requires a total of 2047 events (deaths). Accruing 2200 patients (1100 in each group) over 3 years with a further 1 year follow-up should be sufficient to achieve this required number of events [41]. Through discussions with the UK lung cancer clinical community and patient representatives it was deemed that, balancing the additional expense, risk of toxicity and logistics of giving dalteparin, an increase of 5% in 1 year survival was clinically significant. The current rate of VTE ante mortem is 15%, with a post mortem VTE rate in excess of 50% in advanced cancer. The use of thromboprophylaxis could therefore have a larger impact on survival.

### Method of Randomisation

Patients are randomised centrally by the Wales Cancer Trials Unit using the method of minimisation which includes a random element. Patients are stratified for a number of clinically important stratification factors. The randomisation allocation ratio for control: research arm will be 1:1.

### Outcome Measures

The primary outcome measure is overall survival. Overall survival is calculated from the date of randomisation, to death from any cause. Those patients still alive will be censored at the date last seen.

The secondary outcome measures are: VTE-free survival, metastasis-free survival, toxicity, QoL, dyspnoea, anxiety and depression, cost effectiveness and cost utility.

VTE-free survival will be calculated from the date of randomisation to the date of first clinical evidence of VTE or death from any cause (whichever is the earlier). VTE-free survivors will be censored on the date last known to be alive and free of clinical evidence of a VTE. Metastasis-free survival will be calculated from the date of randomisation to the date of first clinical evidence of metastatic disease or death from any cause (whichever is the earlier) in those patients whose baseline staging indicates no evidence of metastases at the time of randomisation. Metastasis-free survivors will be censored on the date last known to be alive and free of clinical evidence of metastatic disease. Toxicity will be assessed according to NCI Common Terminology Criteria for Adverse Events version 3.0 and any serious adverse events (SAE) are collected in 'real time'. QoL questionnaires will be completed by patients at baseline, 12 weeks, 24 weeks, 9 months and 1 year. QoL will be generally assessed using EQ-5D which includes a core of five domains (mobility, self-care, usual activities, pain/discomfort and anxiety/depression) with three options per domain. A unique EQ-5D health state is defined by combining the scores from each of the 5 dimensions to produce a score from 0 to 1. The impact of dyspnoea (breathlessness) will be assessed using the Cancer Dyspnoea Scale, which consists of twelve items with five options. These items are combined to create three factors that represent the sense of effort, anxiety and discomfort. The impact on mental health (anxiety and depression) will be assessed using the Hospital Anxiety and Depression Scale (HADS), which consists of fourteen items with four options, which are summed to create two subscales 'depression' and 'anxiety'. Cost effectiveness of dalteparin will be assessed. Costs will be monitored prospectively via Case Report Forms (CRF) for 1 year or until death, whichever is soonest. Survival will be adjusted for quality of life using the EQ-5D.

### Data collection

Participants will be seen at hospital every 3-4 weeks until 24 weeks after randomisation, follow up visits will occur at months 9 and 12, and then at yearly intervals. Research staff at the hospitals will be expected to complete trial CRFs which record evidence of primary and secondary outcome measures.

### Statistical analysis

All analyses will be performed on a full intention-to-treat basis, i.e. all patients randomised will be included, and all patients will be analysed according to their allocated group whatever treatment they received. Descriptive statistics of the patient characteristics within each treatment group will be presented (including a summary of the type of standard anticancer treatment each patient actual received). The main analysis will compare overall survival between the two groups using an unadjusted logrank test. Final analysis will take place when 2047 events (deaths) have been reported, which is expected to be approximately 1 year after accrual closes. Kaplan Meier curves and logrank tests will also be used to compare the two groups on the secondary outcomes of VTE-free survival and metastasis-free survival. N.B. It is possible the data for secondary endpoints (e.g. toxicity) may be mature enough for analysis and presentation before the main overall survival primary endpoint. The secondary outcomes of proportions of patients with toxicities and SAEs such as unexplained death and significant bleeding during the first six months will be compared using a chi-squared test. An assessment of compliance with medication in the dalteparin arm will be made, including an exploration of predictors of poor compliance. Secondary analysis will incorporate an assessment of the impact of compliance on overall VTE-free and metastasis-free survival [42]. The analysis of QoL, dyspnoea, anxiety, and depression endpoints will use both a subject-specific and group based approach. The subject-specific approach will use the data for each individual, the severity of each symptom being plotted against the assessment time and then the areas under the curve (AUC) calculated. The AUC is then standardized by dividing by the number of days between the first and last assessment, resulting in a standardized AUC (SAUC) for each patient, for each symptom. The Mann-Whitney test will be used to compare SAUCs between treatment arms. The group based approach considers the proportion of all patients in each treatment group falling into each symptom category over time. The summary of this data gives an impression of the severity of each symptom at each specific time point in the trial and is compared between treatment arms using a chi squared test. The economic evaluation will be in the form of a cost utility analysis from a secondary care perspective. Mean differential costs between groups will be estimated. Since cost data are often skewed, bootstrapping methods will be used to produce 95% confidence intervals alongside point estimates. Probabilistic sensitivity analyses will account for both parameter and decision uncertainty. The EQ-5D allows estimation of quality adjusted life years (QALYs) which will be the main effectiveness measure in the economic analysis. Between group differences will be estimated using the area under the curve method adjusted for differences at baseline. In the case of non-dominance, results will be reported in the form of an incremental cost effectiveness ratio (ICER) which shows the extra cost of producing one extra QALY. A cost-effectiveness acceptability curve (CEAC) will be used to calculate 95% confidence intervals for the incremental cost effectiveness ratios http://www.euroqol.org. No formal subgroup analyses are planned to look at differences in primary and secondary endpoints between treatment groups within specific groupings based on patient characteristics. However exploratory analysis will be conducted to explore whether there is any consistent benefit from using dalteparin in different subgroups by creating Hazard Ratio plots and carrying out tests for interaction/trend based on chi squared analysis.

## Discussion

This Cancer Research UK funded trial will be one of the biggest lung cancer trials ever conducted in the world and will be the first to determine whether the addition of LMWH to routine treatment will be advantages in terms of overall survival in patients with lung cancer.

## Competing interests

None of the authors have any competing interests apart from receiving investigator lead research grants and free drug from Pfizer Inc. for this trial and other WCTU studies.

## Authors' contributions

FRM and SIN were responsible for the research question, design of the trial and contributed to the writing of the study protocol. GOG is the Scientific Director and TSM the Clinical Director of the Wales Cancer trials Unit and have overall responsibility for running the trial at the WCTU. SB is the Trial Manager for the trial, and participated in its design and coordination. DC is the Health Economist for the trial and participated in the study design. All authors have read and approved the final manuscript.

## Pre-publication history

The pre-publication history for this paper can be accessed here:

http://www.biomedcentral.com/1471-2407/9/355/prepub
